# A multimodal concept for vaginal cuff closure by modification of the Bakay technique in total laparoscopic hysterectomy: a randomized clinical study

**DOI:** 10.1186/s12905-021-01591-z

**Published:** 2022-01-08

**Authors:** Üzeyir Kalkan, Kadir Bakay

**Affiliations:** 1 Department of Obstetrics and Gynaecology, Koç University Hospital, Topkapı, Davutpaşa Cd. No: 4, 34010 Zeytinburnu, Istanbul, Turkey; 2 Department of Obstetrics and Gynaecology, Faculty of Medicine, Ondokuz Mayis University, Samsun, Turkey

**Keywords:** Laparoscopic surgery, Hysterectomy, Apical prolapse, Suture technique, Colpotomy

## Abstract

**Background:**

The aim of this study was to compare the outcomes of modified Bakay technique (MT) to standard colpotomy (ST) and cuff closure in total laparoscopic hysterectomy (TLH).

**Methods:**

This two-centre, randomized-controlled study included a total of 160 patients who were scheduled for TLH for benign diseases (ClinicalTrials.gov Identifier is NCT05080114 and the first posted date was 15/10/2021). The patients were allocated into two groups by a computer-based randomization programme as ST group and MT group. Total operative time, cuff closure time, length of hospital stay, intra- and postoperative complications according to the Clavien-Dindo classification, pre- and postoperative vaginal length, and patient satisfaction according to the Patient Global Impression of Improvement (PGI-I) questionnaire were assessed.

**Results:**

Seventy-seven patients in the ST group and 80 patients in the MT group underwent TLH. The total operative time was significantly shorter in the MT compared to the ST (55.5 vs. 59 min, respectively; *p* = 0.001). The median total operative time for colpotomy, extraction of uterus, and vaginal cuff closure steps was 9 (range 6–12 in MT vs. 6 to 11 in ST) min in both groups. The median hospital stay was 2 (range 1–4) days in both groups. Intraoperative blood loss was not significantly different between the groups (90 mL in ST vs. 80 mL in MT; *p* = 0.456). The mean uterine weight for the ST group and MT group was comparable (258.6 ± 88.6 g vs. 232.9 ± 102.5 g, respectively; *p* = 0.107). The preoperative vaginal length was not significantly different between the groups (*p* = 0.502). The median postoperative vaginal length was significantly higher in the MT group compared to the ST group on Day 90 (8 cm vs. 7,5 cm, respectively; p = 0.001). The PGI-I questionnaire score on Day 90 postoperatively was 2 (range 1–5) in both groups (*p* = 0.636). The complication rates were similar between the groups (*p* = 0.230).

**Conclusion:**

The MT can be safely performed in most of the cases requiring TLH with the advantages of vaginal cuff closure before the alteration of pelvic anatomy, support to primary healing of the vaginal cuff, and routine concomitant apical support.

**Supplementary Information:**

The online version contains supplementary material available at 10.1186/s12905-021-01591-z.

## Introduction

Although the benefits of laparoscopy including less postoperative discomfort, short recovery time, and improved patient outcomes are well documented in the literature [1], there are many factors still limiting the wide acceptance and implementation of the technique for hysterectomy [2]. The most challenging steps of total laparoscopic hysterectomy (TLH) are colpotomy and cuff closure. The basis for minimizing the rate of severe haemorrhage and ureteral injuries, the most serious events related to these steps, is meticulous dissection providing a clear operative field and the skill and experience of the surgeon. In TLH, the altered anatomy after the removal of the uterus may cause the retraction of vagina and shifting of neighbouring structures such as bladder and/or bowel to this pouch, thereby, leading to obstruction of the operative field for vaginal cuff closure [3, 4].

Electrosurgical colpotomy is usually the preferred technique in TLH. However, this may cause more extensive tissue necrosis and devascularization, leading to a thicker inflammatory infiltrate and late tissue healing (i.e., per secundam intentionem). This prolonged inflammatory phase may increase the risk of cuff cellulitis, dehiscence, granulation and secondary haemorrhages. Therefore, it is of utmost importance to put the sutures beyond the demarcation line of this thermal damage for tissue integrity [5].

The apical uterovaginal support is compromised by total hysterectomies [6]. Unless re-attachment of the uterosacral ligament (USL) complex to vaginal cuff is performed, Level I support is lost, potentially leading to future vaginal vault prolapse [7, 8]. A concomitant procedure for apical support performed during hysterectomy lowers the re-operation rates for pelvic organ prolapse in the future [9]. Despite this, apical support procedures appear to be underutilized: they are performed in only 3% and 55% of cases without and with a diagnosis of uterovaginal prolapse [10]. The American Association of Gynecologic Laparoscopists (AAGL) also recommends USL suspension at the time of TLH to prevent future vaginal vault prolapse [11].

To date, various methods of cuff closure in TLH have been described [4, 12, 13]. Bakay [14] published his novel colpotomy and cuff closure technique for TLH in 2018. This study was the first to describe placing a single continuous running purse-string suture facilitating the cuff closure before colpotomy. The main advantage of the technique involved retrieving the safe suture margins required for vaginal cuff closure before the pelvic anatomy was altered by the removal of the uterus. In addition to this advantage, we modified the technique to achieve a better cuff healing and standardized apical support and the modified Bakay technique (MT) proposes: (i) placing a single continuous running purse-string suture for vaginal cuff closure before the pelvic anatomy is altered by the colpotomy and removal of the uterus; (ii) suspension/plication of USLs (as a well-defined, efficient, concomitant apical support procedure to prevent future vaginal vault prolapse) routinely in each case before colpotomy while the margins of these ligaments and adjacent structures such as ureters are still prominent and pelvic anatomy is not altered; and (iii) using cold-knife colpotomy instead of electrosurgical colpotomy to support the primary healing of the vaginal cuff. In the present study, we aimed to compare the surgical and clinical outcomes of the MT to standard technique (ST) in patients undergoing TLH.

## Materials and methods

### Study design and study population

This two centre, randomized clinical study was conducted at Departments of Obstetrics and Gynaecology of two tertiary care centres between November 2018 and September 2020. The patients who were scheduled to undergo TLH for benign diseases were screened. Pelvic examination, transvaginal ultrasound, Pap-smear and endometrial biopsy, urine analysis, blood analysis including complete blood count, prothrombin time and partial thromboplastin time, and electrocardiography were performed preoperatively. Prior to study, all patients were informed about the nature of the study. A written informed consent was obtained from each patient. The study protocol was approved by the institutional Science and Ethics Committee (Application Date: 23.02.2018 and Approval No: OMU KAEK 2018/22). The study was conducted in accordance with the principles of the Declaration of Helsinki. ClinicalTrials.gov registration number is NCT05080114 and the first posted date was 15/10/2021.

In each centre, patients were randomized using an online-generated block randomization sequence with allocation to undergo either TLH with MT (n = 40) or ST (n = 40) in a 1:1 ratio design performed via a series of sealed opaque envelopes. As a sum of two centres, MT and ST groups included 80 patients each. The surgeon was blind to the allocation until the steps leading to colpotomy were completed during the surgery. At this point, the coordinator nurse opened the sealed opaque envelope and declared the type of technique to be proceeded. Patients were blinded to the allocation until the postoperative visit on day 90 was completed. The main indications for surgery were symptomatic uterine fibromatosis, chronic pelvic pain, simple or complex endometrial hyperplasia, menorrhagia/adenomyosis, or benign adnexal masses. Patients with anaesthetic contraindications to laparoscopy, premalignant or malignant genital disease, prior pelvic and/or abdominal radiotherapy, large adnexal masses; large fibroids obscuring the visualization of the cervicovaginal junction; suspicion of malignancy; and pelvic organ prolapse Stage > 2 were excluded. All procedures were performed by two gynaecologic surgeons who are both skilled and experienced in laparoscopic surgery.

### Definitions and outcome measures

The operative time was defined as the time interval between the placement and removal of the primary trocar. As the sequence of colpotomy, removal of uterus and cuff suturing steps are different in each technique, operative times of colpotomy, extraction of uterus, and vaginal cuff closure steps were each added for comparison. Blood loss was measured by examining the amount of blood aspirated during the procedure.

Preoperative vaginal length measurements were taken during the first preoperative vaginal examination in the relaxed dorsal lithotomy position by wooden tongue depressors scaled before. The distance from posterior vaginal vault to hymen was recorded as the vaginal length. Postoperative vaginal length measurements and Patient Global Impression of Improvement (PGI-I) questionnaire [15] was performed on Day 90 postoperatively. The gynaecologic examination and vaginal length measurement on day 90 was performed by another gynaecologist who was non-involved in the study and blind to the type of technique performed. The PGI-I questionnaire consisting of the question “How is your postoperative condition now, compared to how it was before you had the surgery?” was filled by the patient in the waiting room before the pelvic examination on Day 90. The response for the question ranges from 1 to 7 with lower scores indicating a greater level of satisfaction.

Postoperative complications were recorded during follow-up visits on Day 7, 30, and 90. Classification of complications was made based on the standardized Clavien-Dindo reporting system [16].

### Surgical procedure

All operations were performed under general anaesthesia with nasogastric intubation and a bladder catheter in place. Cefazolin 2 g was administered to all patients for prophylaxis 30 min prior to surgery. Operations were performed with a 10-mm laparoscope (Karl Storz, Germany) through the trocar placed usually in the umbilicus. Two lateral 5-mm trocars and one midline 10-mm trocar were used. The placement of trocars varied according to the uterine size. Haemostasis was usually performed using bipolar forceps (Karl Storz Robi, Germany), whereas dissection was performed using the LigaSure™ (Covidien, Medtronic, USA) Maryland jaw laparoscopic sealer/divider, bipolar forceps and scissors. In both groups, all the steps of the TLH leading to colpotomy were performed according to standard protocol [17]. The summary of the steps of standard protocol were as follows:

1. After the visual inspection of the ureter at the pelvic brim, the infundibulopelvic ligaments or, utero-ovarian ligaments and round ligaments were coagulated and incised.
2. The bladder flap was incised, and the anterior cervical fascia was exposed with blunt dissection off of the cervix broadly below the cervicovaginal margin.
3. The uterine arteries were coagulated, cut and pushed downward to expose the cardinal ligament fibers attaching the arteries to the cylindrical cervical fascia; then the cardinal fibers were incised posteriorly to the uterosacral ligaments, and inferiorly, identifying the cervicovaginal margin as the lowest limit of dissection.
4. The colpotomy was performed at the precise margin of the cervix and vagina.
5. After the uterus was removed, the vaginal apex was closed with a 0 Monocryl™ violet (poliglecaprone 25) or a 0 PDS II (polydioxanone) suture with a 36-mm needle (Ethicon Inc., NJ, USA) in 3 or more intracorporeal spiral sutures.

**Fig. 1:**
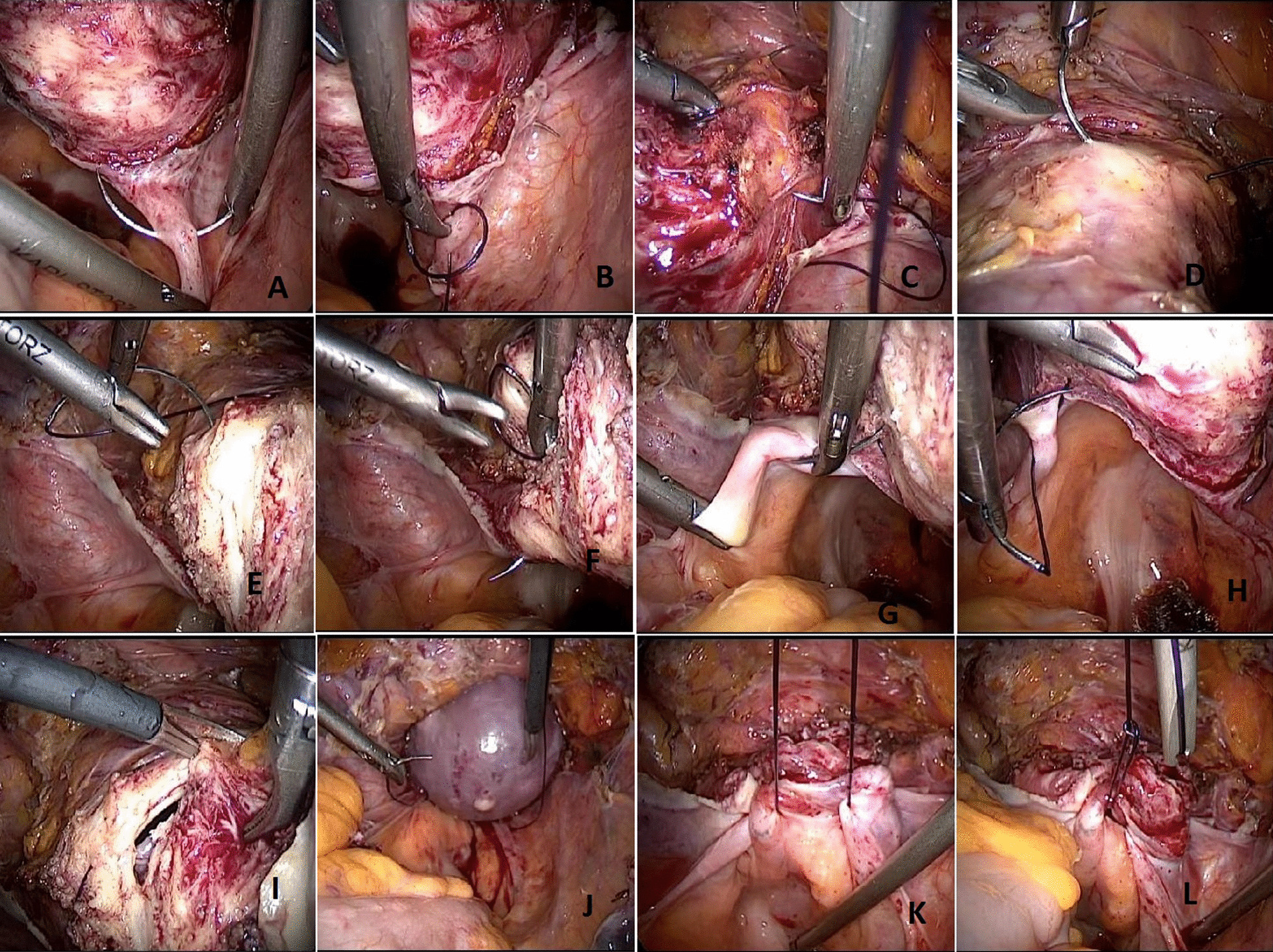
The steps of the modified Bakay technique are described. First suture is placed on the right USL (**A**) then helically proceeded for suspension/plication (**B**). The suture continued circumferentially in the counterclockwise direction on the line between the cervicovaginal junction and the bladder (**C**–**E**) in a full-thickness purse string fashion. This suture symmetrically ended in the left USL, across the point of the start (**F**–**H**). Colpotomy is performed circumferentially using laparoscopic cold scissors and/or knife (**I**), maintaining a safe distance from the suture line, paying attention not to cut the suture. The detached uterus is removed vaginally (**J**) and both ends of the prior suture line are knotted securely (**K**, **L**)

However, the sequence and technique of the colpotomy and cuff-closure steps in the MT group were different as follows:

1. A 0 Monocryl™ violet (poliglecaprone 25) or a 0 PDS II (polydioxanone) suture with a 36-mm needle (Ethicon Inc., NJ, USA) through the 10-mm midline trocar was placed first on the right USL, proximal to the ischial spine, usually 1 to 3 cm away from its uterine insertion, then helically proceeded by 1 to 3 bite(s) (depending on the length of the ligament) for suspension/plication. The suture continued circumferentially in the counterclockwise direction on the line between the cervicovaginal junction and the bladder in a full-thickness purse string fashion, paying attention not to get closer than 1 cm to the bladder. This suture symmetrically ended in the left USL, across the point of the start, again by completing the last bite(s) helically for suspension/plication, forming nearly an Ohm sign (Ω). Once accomplished, suture ends were pulled outside by the assistant surgeon via the10-mm port for retraction and further extracorporeal knot tying; or left inside and grasped by an instrument for further intracorporeal knot tying. This gentle retraction was also intended to slightly pull the suture ends in case of a bleeding that might occur during the cold scissor/knife colpotomy.
2. Colpotomy was performed circumferentially using laparoscopic cold scissors and/or knife, maintaining a safe distance from the suture line, paying attention not to cut the suture.
3. The detached uterus was, then, removed vaginally and both ends of the prior suture line were knotted securely. The steps are summarized in Fig. 1. The closed vaginal cuff was manually examined for any defects under endoscopic visualization. No additional sutures were used.

The first video (Additional file 1: Video 1) shows the procedure of a case with no history of abdominal operations. The second video (Additional file 2: Video 2) shows a case with a history of caesarean section.

All patients were informed to refrain from sexual intercourse for at least 60 days after the operation.

### Main outcomes

Demographic and clinical data, history of previous surgery, and intra- and postoperative findings were recorded. Operative time (time interval between the placement and removal of primary trocar); total operative time for colpotomy, extraction of uterus, and vaginal cuff closure steps; uterine weight (measured before immersion in formaldehyde); blood loss (estimate of the fluid aspirated during the procedure); and length of hospital stay were also noted. The complications were recorded by the clinical evaluation of the patients on Day 7, 30, and 90 postoperatively. Pre- and postoperative vaginal length and patient satisfaction were evaluated. The main outcomes of both groups were compared.

### Statistical analysis

Statistical analysis was performed using the SPSS version 23.0 software (IBM Corp., Armonk, NY, USA). Descriptive data were presented in mean ± standard deviation (SD), median (min–max) or number and frequency (Clavien-Dindo classification of intraoperative and postoperative complications), where applicable. Normal distribution of variables was tested using the Kolmogorov–Smirnov test. Independent samples *t*-test was used to compare normally distributed data (uterine weight), while the Mann–Whitney U was used to compare data that did not show normal distribution (age, BMI, parity, operative time, intraoperative blood loss, lenght of hospital stay, total operative time for colpotomy + extraction of uterus + vaginal cuff closure, preoperative vaginal length, postoperative vaginal length, patient satisfaction by PGI-I scale on day 90 postoperatively). The chi-square test was used for the comparison of categorical data (history of pelvic surgery, indications for hysterectomy, menopausal status, intraoperative and postoperative complications). A *p* value of < 0.05 was considered statistically significant.

## Results

**Fig. 2:**
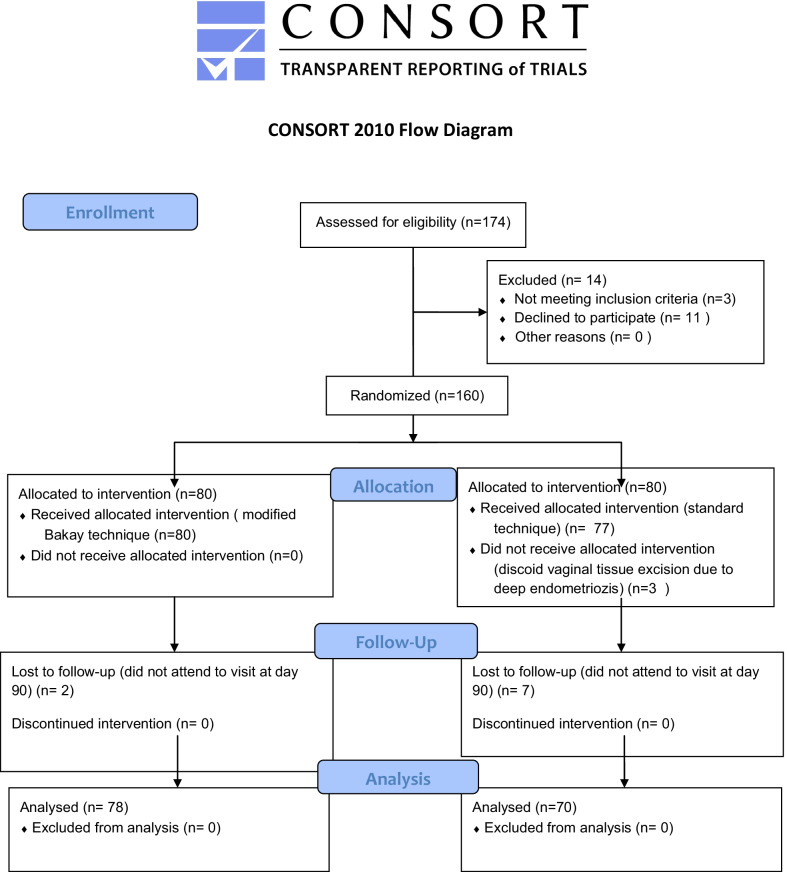
The CONSORT study flow chart

A total of 174 patients scheduled to undergo TLH for benign diseases were screened. Eleven patients refused to participate and three patients were excluded due to anaesthetic contraindications to laparoscopy. Finally, 160 patients who met the inclusion criteria were allocated into two groups by a computer-based randomization programme as ST group and MT group. Of 80 patients allocated for ST group, 10 patients were dropped out (n = 7 not attending to the visit on Day 90, n = 3 having a diagnosis of deep infiltrating endometriosis requiring discoid vaginal wall resection during TLH). Among 80 patients allocated for MT group, only two patients were dropped out, as they did not attend to visit on Day 90. Finally, a total of 70 patients in the ST group and 78 patients in the MT group were included in the analysis. The CONSORT study flow chart [18] is shown in Fig. 2.

Age, body mass index (BMI), parity, menopausal status, history of pelvic surgery, and indications for laparoscopic surgeries did not differ significantly between the groups (Table 1). There were no conversions to laparotomy in either group.

**Table 1:** Demographic data of the patients and indications for surgery

	Standard technique group (n = 70)	Modified Bakay technique group (n = 78)	Total (n = 148)	*p* value
Age (year)	46 (35–56)	47 (36–59)	47 (36–59)	0.826^a^
BMI (kg/m^2^)	28.1 (19.2–40.6)	26.8 (19.2–42.1)	27.4 (19.2–42.1)	0.763^a^
Parity	2 (0–5)	2 (0–5)	2 (0–5)	0.291^a^
**History of pelvic surgery**				
Yes	35 (50)	45 (57.7)	80 (54.1)	0.348^b^
No	35 (50)	33 (42.3)	68 (45.9)	
**Indications for hysterectomy**				0.786^b^
Menorrhagia and Adenomyosis	21 (30)	17 (21.8)	38 (25.7)	
Myoma	31 (44.3)	34 (43.6)	65 (43.9)	
Endometrial polyp	5 (7.1)	7 (9)	12 (8.1)	
Adnexal mass	6 (8.6)	11 (14.1)	17 (11.5)	
Chronic pelvic pain	3 (4.3)	5 (6.4)	8 (5.4)	
Simple or complex hyperplasia	4 (5.7)	4 (5.1)	8 (5.4)	
**Menopausal status**				0.549^b^
Premenopause	49 (70)	51 (65.4)	100 (67.6)	
Postmenopause	21 (30)	27 (34.6)	48 (32.4)	

^a^Mann Whitney U test, ^b^Chi-square test

Operative data and outcome measures are shown in Table 2. The median operative time for ST group was 59 (range, 50 to 75) min and 55.5 (range, 34 to 80) min for the MT group. The operative time was significantly shorter in the MT group (*p* = 0.001). The median total operative time for colpotomy, extraction of uterus, and vaginal cuff closure was equal in both groups (9 min for both groups). In both groups, the median length of hospital stay was 2 (range, 1 to 4) days. The median intraoperative blood loss was 90 mL in the ST group and 80 mL in the MT group, indicating no statistically significant difference (*p* = 0.456). The mean uterine weight for the ST group and MT group was 258.6 ± 88.6 g and 232.9 ± 102.5 g, respectively, indicating no statistically significant difference (*p* = 0.107). The median preoperative vaginal length was not significantly different between the groups (*p* = 0.502). However, the median postoperative vaginal length on Day 90 was significantly higher in the MT group compared to the ST group (8 [6–9.5] cm vs. 7.5 [5.5–9] cm, respectively; *p* = 0.001)). The median patient satisfaction as measured by PGI-I scale on Day 90 was 2 (range 1–5) in both groups, indicating no statistically significant difference (*p* = 0.636).

**Table 2:** Surgical data and outcome measures

	Standard technique group (n = 70)	Modified Bakay technique group (n = 78)	Total (n = 148)	*p* value
Operative time (min)	59 (50–75)	55.5 (34–80)	57 (34–80)	0.001^a^*
Intraoperative blood loss (ml)	90 (60–150)	80 (60–180)	90 (60–180)	0.456^a^
Lenght of hospital stay (d)	2 (1–4)	2 (1–4)	2 (1–4)	0.949^a^
Uterine weight (g)	258.6 ± 88.6	232.9 ± 102.5	245 ± 96.7	0.107^b^
Total operative time for colpotomy, extraction of uterus, and vaginal cuff closure (min)	9 (6–11)	9 (6–12)	9 (6–12)	0.441^a^
**Intraoperative and postoperative complications**				
Yes	6 (8.6)	3 (3.8)	9 (6.1)	0.230^c^
No	64 (91.4)	75 (96.2)	139 (93.9)	
Preoperative vaginal length (cm)	8 (6.5–10)	8.5 (6.5–10)	8 (6.5–10)	0.502^a^
Postoperative vaginal length (cm)	7.5 (5.5–9)	8 (6–9.5)	7.5 (5.5–9.5)	0.001^a^*
Patient satisfaction by PGI-I scale on day 90 postoperatively	2 (1–5)	2 (1–5)	2 (1–5)	0.636^a^

^a^Mann Whitney U test, ^b^Independent samples t test; ^c^Chi-square test

^*^p value < .05

The complication rates were not significantly different between the groups (*p* = 0.230). There was one bladder injury, one urinary tract infection and one trocar site infection in each group. Bladder injuries were detected and repaired intraoperatively. The trocar site and urinary infections were successfully treated with proper antibiotherapy. In the ST group, additionally, one patient had fever (> 38 °C) who was successfully treated with antibiotic and antipyretics, one patient had ileus who was successfully treated with conservative measures such as oral restriction, antiemetics, and electrolyte solution infusions, and one patient had cuff granulation who was diagnosed on Day 60 and treated by electrocoagulation under local anaesthesia. Complications are listed in Table 3.

**Table 3:** Intraoperative and postoperative complications

	Clavien-Dindo classification	Standard technique group (n = 70)	Modified Bakay technique group (n = 78)	Total (n = 148)
**Intraoperative and postoperative complications (%)**				
Bladder injury	Grade 3b	1 (1.4)	1 (1.3)	2 (1.4)
Urinary tract infection	Grade 2	1 (1.4)	1 (1.3)	2 (1.4)
Trocar site infection	Grade 2	1 (1.4)	1 (1.3)	2 (1.4)
Fever	Grade 2	1 (1.4)	0	1 (0.7)
Ileus	Grade 1	1 (1.4)	0	1 (0.7)
Cuff granulation	Grade 3a	1 (1.4)	0	1 (0.7)

## Discussion

The main outcomes of the study revealed a shorter operative time; higher postoperative vaginal length; similar complication rate and patient satisfaction for MT compared to ST.

Various techniques for vaginal cuff incision and closure in TLH have been described in the literature [4, 12, 13]. In this study, the Bakay technique [14] was modified to achieve a routine concomitant effective apical support to prevent future vaginal vault prolapse, and primary healing of the vaginal cuff by using cold-knife colpotomy, instead of electrosurgical colpotomy, in addition to the former objective of the technique that was vaginal cuff closure with safe suture margins before the pelvic anatomy was altered by the removal of the uterus. The clinical and surgical outcomes of the MT showed successful results compared to ST.

Colpotomy and cuff closure steps are technically the most difficult and prolonged parts of TLH. Additionally, the most frequent complications of TLH, which are ureteral injury and bleeding, mostly occur in these steps. One of the main advantages of the MT involves placing the sutures before the pelvic anatomy is altered by the colpotomy/removal of the uterus providing to maintain the required suture margins with a safe distance from the bladder, ureters, and bowels in a shorter total operating time.

To the best of our knowledge, there is no study specifically comparing cold-knife colpotomy with electrosurgical colpotomy at TLH. During the colpotomy, monopolar current when applied at 60 W for 1 s exhibits a mean critical spread of 3.5 mm; however, the spread exceeds 20 mm when applied for ≥ 2 s [19]. Thus, a more prolonged continuous current may increase the extent of collateral damage. Current recommendations for colpotomy and cuff closure in TLH are to minimize lateral thermal spread to maintain tissue integrity and to place sutures well beyond the border of thermal damage to prevent vaginal cuff dehiscence and evisceration [20]. In MT, using cold-knife colpotomy, instead of electrosurgical colpotomy, we intended to eliminate extensive tissue necrosis and devascularization leading to a thicker inflammatory infiltrate and late tissue healing (per secundam intentionem). As a point of good practice, this may contribute to prevent cuff cellulitis, cuff dehiscence, and secondary cuff haemorrhages by supporting the primary healing of the vaginal cuff. Also, our technique may tolerate the smaller purchases on vaginal cuff being sutured caused by the laparoscopic magnification of the surgical field. In this study, there was no significant difference in the postoperative complication rates between the groups, although the sample size is too small to specifically compare the rate of complications such as cuff cellulitis, dehiscence, granulation, and secondary cuff haemorrhages. This may be the subject of a future comparative study with a larger sample size.

In the current study, the total vaginal length was well preserved by our technique which may be important for sexual function. The MT was associated with longer postoperative vaginal length, compared to the ST which can be attributed to a better cuff healing achieved by cold colpotomy that prevents the loss of the vaginal tissue demarcated by thermal damage. In the literature, there are few studies investigating the effect of vaginal length on sexual function and showing a weak correlation measured by Female Sexual Function Index questionnaire [21–23]. However, the evaluation was usually done shortly after hysterectomy (i.e., three months). This may be a distressing factor negatively contributing on sexual function. Thus, assessment of the sexual function in the long-term may eliminate this factor. Of note, the effect of our technique on sexual function was not the scope of our study due to short-term evaluation and small sample size.

It is evident in the literature that omitting a concomitant apical support procedure during hysterectomy increases the re-operation rates for pelvic organ prolapse in the future [9]. Despite this, apical support procedures are not performed in most of the cases without uterovaginal prolapse. Even in cases with uterovaginal prolapse, only half of them receive a concomitant apical support procedure [10]. Due to this underutilization, the AAGL recommends USL suspension at the time of TLH to prevent future vaginal vault prolapse [11]. In our technique, suspension and/or plication of USLs was a routine step, leading to concomitant support of vaginal apex to prevent future vaginal vault prolapse. The procedure could be safely accomplished, while the margins of USLs and adjacent structures such as ureters were still prominent and pelvic anatomy was not altered. The number of suture loops depended on the prominent length of USLs (usually 1–3 bites). Thus, at least with only one bite, USL suspension was possible, while USL plication could be also performed with two or three bites.

In the present study, the total operative time for colpotomy, extraction of uterus, and vaginal cuff closure were not significantly different between the groups, although the MT was associated with shorter total operative time compared to the ST. When the videos of the procedures were revisited, we found that the time loss leading to this difference was mostly related to checks for reassuring the safe margins to the bladder or demarcation line of thermal damage and haemostasis control for small bleedings in folded areas around the cuff and parametrium after cuff closure. Using the MT, placing the sutures before the pelvic anatomy was altered, slight retraction of both suture ends preventing unwanted bleedings during the colpotomy and cold colpotomy was probably eliminating the need for further checks, thereby, shortening the total surgical time.

One bladder injury was reported in each group which is consistent with the literature [24]. Both injuries occurred in patients with prior pelvic surgery. The rate of bladder injury ranges from 0.2 to 1.8% in the literature and is mostly associated with previous laparotomy [24]. In contrast to prior study [14], unintentional cutting of the suture line during the cold colpotomy which increases the operative time did not occur in this study. This probably was due to experience gained by the increased number of cases. Also, a limiting factor for the Bakay technique was the size of uterus, as it was indicated in the previous study [14]. Although there was no statistically significant difference between the groups in terms of uterine weight, we analysed the data to determine the largest uterus removed by ST and MT in our study. The results were 465 g and 510 g, respectively, indicating that gaining experience in this technique could overcome the limitations described in the previous study- to some extent- related to large uterine size due to inadequate visualization of the posterior area.

In our study, there was no significant difference in terms of postoperative complication rates and patient satisfaction scores between the groups at three months after the operations. In both groups, the patients described their postoperative condition as “much better” compared to preoperative condition.

Nonetheless, there are some limitations to this study. The ancillary port localizations in this study are symmetrical (contralateral). Therefore, a possible difficulty for circular suturing of the cuff for surgeons working with ancillary trocars ipsilaterally may exist until they adapt to the technique. It is not possible to reach a conclusion related to the effects of the MT on pelvic organ prolapse with a short term (90 days) follow up. Long-term outcomes of the MT related to cuff healing and pelvic organ prolapse should be evaluated in future prospective studies with adequate sample sizes. Although the sample size of this study is adequate to test most of the operative data, larger studies are needed to evaluate all possible complications of the novel technique. Further multi-centre, large-scale studies involving multiple number of surgeons are warranted to test the applicability and adoption of the technique in the surgical practice. Also, a possible difficulty for circular suturing of the cuff for surgeons working with ancillary trocars ipsilaterally may be considered.

## Conclusion

In conclusion, the MT can be safely utilized in most of the cases requiring TLH with certain advantages of vaginal cuff closure before the alteration of pelvic anatomy, support to primary healing of the vaginal cuff and routine concomitant apical support, even in some cases with a large uterus or history of pelvic surgery.

## Supplementary Information

**Additional file 1: Video 1.** Video showing the modified Bakay procedure of a case with no history of abdominal operations.

**Additional file 1: Video 2.** Video showing the modified Bakay procedure of a case with a history of caesarean section.

## Data Availability

The data that support the findings of this study are available at Ondokuz Mayıs University and Egemed Hospitals database, which are not publicly available. Data are available from the authors upon reasonable request and with permission of the Ondokuz Mayıs University and Egemed Hospitals.

## Abbreviations

TLH: Total laparoscopic hysterectomy
USL: Uterosacral ligament
MT: Modified Bakay technique
ST: Standard technique
PGI-I: Patient Global Impression of Improvement
